# Iodoformagen

**Published:** 1899-04

**Authors:** 


Iodoformagen. The *Berliner klin. Wochenschrift* says that this substance has come into extensive use on the continent as a substitute for iodoform. It is a compound of iodoform and albumin, and consists of a fine, dry, yellowish powder, readily soluble in water. It is quite free from the disagreeable smell of iodoform and very economical in use, its weight being less than one-third that of ordinary iodoform; and, owing to its solubility and practical freedom from smell, it possesses great advantages over ordinary iodoform. It has been very favorably reported on by leading medical practitioners in Germany.

